# Retraction: Effect of weekly long-acting growth hormone replacement therapy compared to daily growth hormone on children with short stature: a meta-analysis

**DOI:** 10.3389/fendo.2023.1220345

**Published:** 2023-06-09

**Authors:** Frontiers Editorial Office

The journal and Chief Editors retract the 29 November 2021 article cited above.

Following publication, concerns were raised regarding abnormal similarities with the contents of other articles published by unrelated research groups. A subsequent investigation, which was conducted in accordance with Frontiers’ policies, raised strong concerns over the authorship of the articles, resulting in a loss of confidence in the findings presented in the article.

The authors have not responded to this retraction.

This retraction was approved by the Chief Editors of Frontiers in Endocrinology and the Chief Executive Editor of Frontiers.

